# Managing ecological thresholds of a risky commons

**DOI:** 10.1098/rsos.230969

**Published:** 2023-10-18

**Authors:** Sukanta Sarkar

**Affiliations:** Odum School of Ecology, University of Georgia, Athens, GA 30602, USA

**Keywords:** evolutionary game theory, replicator dynamics, critical transitions, cooperation, common resource

## Abstract

Common resources are often overexploited and appear subject to critical transitions from one stable state to another antagonistic state. Many times resulting in tragedy of the commons (TOC)—exploitation of shared resources for personal gain/payoffs, leading to worse outcomes or extinction. An adequate response would be strategic interaction, such as inspection and punishment by institutions to avoid TOC. This strategic interaction is often coupled with dynamically changing common resources. However, effect of strategic interaction in complex, coupled socio-ecological systems is less studied. Here, we develop replicator equations using evolving games in which strategy and common resources co-evolve. We consider the shared commons as fish dynamics governed by the intrinsic growth rate, predation and harvesting. The joint dynamics exhibit an oscillatory TOC, revealing that institutions need to pay special attention to intrinsic growth rate and nonlinear interaction. Our research shows that the co-evolving system exhibits a broader range of dynamics when predation is present compared to the disengaged fishery system. We conclude that the usefulness, chances and challenges of modelling co-evolutionary games to create sustainable systems merit further research.

## 1. Introduction

Multistability in ecological systems describes how ecosystems with different initial conditions can stabilize at very different asymptotic equilibriums. Multistable (MS) systems occur in a wide range of dynamic systems, such as coral reefs [1], fisheries [2] and shallow lakes [3]. If an MS system moves past a threshold or tipping point and into a different basin of attraction, a sudden, costly, irreversible transition can occur [4,5]. In particular, overexploitation of a shared resource is a rudimental conundrum that can be discovered in various ecological systems [6,7]. Management and sustainability of MS systems have received much attention in recent years [8,9] in response to financial market crash [10], climate change [11], and vegetation changes [12].

Human-affected MS ecological systems are often viewed as coupled socio-ecological systems (SESs) where human actions influence ecological variables that in turn influence humans [13,14]. As humans have increasingly affected ecological systems, the effects of ecological variables on humans have also grown. Consequently, cognizance of the aftermath increases, and efforts are made to alleviate them. Coupled SESs are, therefore, omnipresent and arise in various systems, common pool resource, agriculture, water research [15], and global climate systems [16].

SES management is often modelled using the evolutionary game theory that combines imitation dynamics or feedback evolving game with ecological modeling [17,18]. Public opinion and conservation are often regarded as coupled SESs in forest systems. SES management refers to altering the stability landscape or topology of the dynamic system (i.e. its basin of attraction or shape-defining thresholds) to achieve a preferred outcome [14,19]. Work on MS SESs includes a foresighted manager or governing body, which guides the system ahead on a comprehensible path towards a steady outcome [20]. Managers will modify actions to achieve consistent and sustainable results across various initial ecological conditions. In such systems, sudden shifts can be averted until the costs exceed the benefits.

Growing research interest shows awareness of developing impregnable fishery regulation policies to prevent fish stocks’ sudden and irreversible decline due to changing environmental and economic pressures. A method of sustainable use of a shared resource could be to penalize those who overharvest and violate the agreed-upon rules [21–26]. Some other frameworks, like public opinion and conservation, and ostracism or volitional pursuance, are also discussed as feasible solutions to the problem of over-exploitation [27,28]. Observational studies, governmental surveys and historical accounts in various countries have noted that conservation increased forest cover [20,29,30]. Importantly in forest systems, inspection, punishment and permanent monitoring mechanisms are used for forest management [31], but still less explored for recreational fishing. Specifically, how higher-order interaction, such as predation in the case of fisheries, affects the dynamics largely remains less studied.

Our primary focus in this study is to investigate how social mechanisms, specifically inspection and punishment, impact the socio-ecological model of fishing. In particular, how inspection and punishment mechanisms influence the complex dynamics of fisheries when the benefit of strategies depends on fish density [18,32]. First, we explore the temporal dynamics of a socio-ecological fish population model for various harvesting rates. Then we developed a coupled co-evolutionary system of fish populations with feedback-evolving games and strategies where the fish population is influenced by feedback of individual strategies and obtained the corresponding stochastic co-evolutionary model using the Markov process of birth and death. We then demonstrate the complex dynamics of the co-evolving system at various intrinsic growth rates. When the intrinsic growth rate is moderate, and inspection and punishment are low, an oscillatory tragedy of the commons occurs. Our results indicate that punishment and permanent monitoring can prevent the overexploitation of fisheries. In addition to the proposed strategies, our study has also revealed that the growth rate and higher-order interaction of the fish population are fundamental for maintaining sustainable fisheries.

## 2. Model and results

### 2.1. Common resource dynamics model

We consider a coupled human environment fish-biomass model as common resource where fish biomass *n* grows logistically to its carrying capacity *k*, but is also harvested:

2.1 $$\frac{dn}{dt}=rn\left(1-\frac{n}{k}\right)-\frac{\;p_{1}n^{2}}{h^{2}+n^{2}}-Hn,$$

where *r* is the net growth rate, *p*_1_ is the predation rate, *h* is the value of *n* where predation is half maximum, and *H* represents harvests by humans. The system (2.1) is a classical overharvesting model [18,32,33]. We refer to *H* as the control variable. Here, we assume that the shared common dynamics is partly renewable, for the limited resource in nature [31]. The system has three equilibria corresponding to no fish density (no resource), stable fish density and unstable fish density.

**Figure 1.**
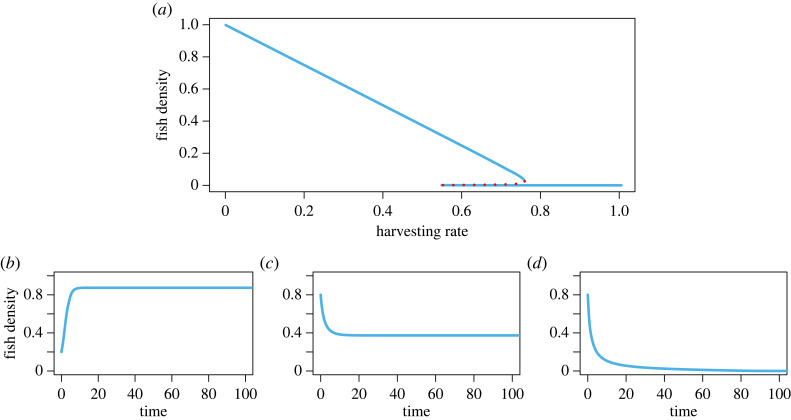
Recreational fishery collapse in the common resource dynamics model. (*a*) Bifurcation diagram of the risky commons for changes in *H*. Solid lines (cyan) represent stable steady states and dotted line (red) represents the unstable steady state. Time series of the common resource for three different values of harvesting efficiency: (*b*) *H* = 0.1, (*c*) *H* = 0.5 and (*d*) *H* = 0.8. Parameters are *r* = 0.8, *p*_1_ = 0.39, *h* = 1, *k* = 1000.

This shared resource model exhibits regime shift due to overharvesting through two saddle-node bifurcations. Bifurcation diagrams are the most effective qualitative tool to comprehend regime shifts, which show the change in equilibrium as the parameter of a system varies. A bifurcation in a dynamical system occurs due to changes in the stability of an equilibrium state. Here, we observe that the system exhibits monostability and bistability (figure 1*a*). With the increase of the harvesting parameter, the system exhibits a critical transition/tipping from a high-density state to a low-density state. With low and moderate harvesting efficiency, the system persists in a stable fish density state (*n* > 0) (depending on the initial condition) (figure 1*b*,*c*), and with high harvesting efficiency, it collapses (figure 1*d*).

### 2.2. Behavioural model with feedback-evolving games

We model the harvests as feedback responses to resources, where responses are conditioned to harvest choices and derived from a decision model of a feedback-evolving game. Furthermore, individuals receive a specific amount from the shared commons to use the resource properly based on the current total resource count *n*. We assume that the amount harvesters get from the commons is *b*_*c*_ = *b*_*m*_*n*/*k*, where harvesters can take a maximum amount *b*_*m*_ from the shared resource at a given time. Here, we assume that harvesters have two different strategies and must choose one. Individuals harvest according to the rule and get *b*_*c*_ from the shared resources, called cooperators. The other is called defectors, which neglects the rule of harvesting and harvests more intensively, getting an amount *b*_*d*_ > *b*_*c*_. Here, *b*_*d*_ = *b*_*c*_(1 + *α*); *α* > 0 signifies the severity of defection.

We introduce an organized resource management institution for the utilization of strategies inspection and punishment to evade shared commons exploitation. More specifically, to quantify the defector’s defect in a given time, we have a probability *p*. Overexploitation of shared commons by an individual will result in a *β* (*β* > 0) fine, which is deducted from the individual’s collected payoff. Thus, *p* describes the effectiveness of monitoring, while *β* describes the severity of punishment [34,35].

Due to overharvesting, shared commons are prone to sudden collapse [17,18,36,37]. Using evolutionary game theory, we consider a social dynamics from the perspective of inspection and punishment. Using a replicator equation, we describe the evolution of punishment and inspection strategies in a well-mixed population [38,39]. The governing dynamic replicator equation is

2.2 $$\frac{dx}{dt}=x(1-x)(U_{c}-U_{d}),$$

where *x* denotes the fraction of cooperators and *U*_*c*_ and *U*_*d*_ are utilities of cooperator and defector players, respectively. We assume that the player’s utility depends on the common resource [37,40,41]. Thus we assume the utility for cooperators *U*_*c*_ = *b*_*c*_ and *U*_*d*_ = *b*_*d*_ − *pβ* for defectors.

**Table 1.** Baseline parameters for the co-evolutionary system.

parameter	definition
*r*	shared resource growth rate
*N*	total number of harvesters
*k*	resource carrying capacity
*α*	severity of defection
*β*	severity of punishment
*b*_*m*_	maximum amount of shared resource at a given time
*p*_1_	predation rate
*p*	effectiveness of monitoring
*h*	half-saturation constant

For the co-evolutionary dynamics of shared resource, which considers predation in the form of Holling’s type II and harvesting, the governing equation is given as

2.3 $$\frac{dn}{dt}=rn\left(1-\frac{n}{k}\right)-\frac{\;p_{1}n^{2}}{h^{2}+n^{2}}-N\{b_{c}x+(1-x)b_{d}\},$$

where *N* is the total number of harvesters in the community. Now by substituting the values of utilities into equations (2.1) and (2.2), we have the coupled system as (all the parameters are mentioned in table 1)

2.4a $$\frac{dx}{dt}=x(1-x)\left(\;p\beta -\frac{n}{k}b_{m}\alpha \right)$$

and

2.4b $$\frac{dn}{dt}=rn\left(1-\frac{n}{k}\right)-\frac{\;p_{1}n^{2}}{h^{2}+n^{2}}-N\frac{n}{k}b_{m}\{1+(1-x)\alpha \}.$$

### 2.3. Stochastic dynamics

Stochasticity is prevalent in nature and can induce new and unexpected phenomena in dynamical systems that cannot be understood alone from deterministic ones. To incorporate intrinsic stochastic fluctuations in the model, we developed a stochastic model corresponding to equation (2.4). In particular, we developed a definite form of the master equation considering all the basic birth–death processes associated with the deterministic model and derived the corresponding Fokker–Planck equation from the master equation (electronic supplementary material, appendix §1). The transition probabilities along with all the molecular events that happen for this circuit are described in electronic supplementary material, appendix table S1.

### 2.4. Stochastic formulation

Master equation of a dynamical system is the time evolution of probabilities for Markov process. Here, we develop the master equation for the grand probability *P*(*x*, *n*, *t*), considering all the fundamental processes for the system (2.4). Here *P*(*x*, *n*, *t*) is the probability that there are *x* cooperators and *n* individuals of common pool resource at time *t*. Reaction #1 cooperator growth due to punishment and inspection and its master equation contribution is

$$\frac{dP}{dt}=(x-1)(1-(x-1))p\beta P(x-1,n)-x(1-x)p\beta P(x,n).$$

Reaction #2 is associated with cooperator decay due to more defection and its master equation contribution is

$$\frac{dP}{dt}=(x+1)(1-(x+1))b_{m}\frac{\alpha }{k}P(x+1,n)-x(1-x)b_{m}\frac{\alpha }{k}P(x,n).$$

Reaction #3 is associated with logistic growth of shared commons and it is given as

$$\frac{dP}{dt}=r(n-1)\left(1-\frac{\left(n-1\right)}{k}\right)P(x,n-1)-rn\left(1-\frac{n}{k}\right)P(x,n).$$

Reaction #4 is for the Holling type II response for predation on shared commons and is given by

$$\frac{dP}{dt}=\frac{\;p_{1}{\left(n+1\right)}^{2}}{h^{2}+{\left(n+1\right)}^{2}}P(x,n+1)-\frac{\;p_{1}n^{2}}{h^{2}+n^{2}}P(x,n).$$

Reaction #5 is associated with decay of shared commons and its master equation contribution is

$$\frac{dP}{dt}=Nb_{m}\frac{n+1}{k}P(x,n+1)-Nb_{m}\frac{n}{k}P(x,n).$$

Reaction #6 is associated with shared commons decay due to more defection and its master equation contribution is

$$\frac{dP}{dt}=Nb_{m}\frac{n+1}{k}\alpha (1-x)P(x,n+1)-Nb_{m}\frac{n}{k}\alpha (1-x)P(x,n).$$

Combining all the above relative microstate transition probabilities, we can proceed to determine the master equation for grand probability *P*(*x*, *n*, *t*) (details are in electronic supplementary material, appendix §1):

2.5 $$\frac{\partial P(x,n,t)}{\partial t}=(x-1)(1-(x-1))p\beta P(x-1,n)-x(1-x)p\beta P(x,n)+(x+1)(1-(x+1))b_{m}\frac{\alpha }{k}P(x+1,n)-x(1-x)b_{m}\frac{\alpha }{k}P(x,n)+\;r(n-1)(1-\frac{\left(n-1\right)}{k})P(x,n-1)-rn(1-\frac{n}{k})P(x,n)+\frac{p_{1}{\left(n+1\right)}^{2}}{h^{2}+{\left(n+1\right)}^{2}}P(x,n+1)-\frac{p_{1}n^{2}}{h^{2}+n^{2}}P(x,n)+Nb_{m}\frac{n+1}{k}P(x,n+1)-Nb_{m}\frac{n}{k}P(x,n)+\;Nb_{m}\frac{n+1}{k}\alpha (1-x)P(x,n+1)-Nb_{m}\frac{n}{k}\alpha (1-x)P(x,n).$$

We have simulated numerically this master equation (2.5) with the Gillespie algorithm [42] to get the stochastic trajectory of the system (electronic supplementary material, appendix §2).

### 2.5. Dynamical outcomes in low growth rate

For our analysis, we first define two quantities, *g*_*c*_ = *N b*_*m*_/*k* and *g*_*d*_ = (*N b*_*m*_/*k*)(1 + *α*), respectively representing cooperators’ and defectors’ gain from a shared commons. From the definition, we have *g*_*c*_ < *g*_*d*_ and *g*_*c*_ > 0. To see the dynamics of the coupled system, we consider three different parameter regions for the intrinsic growth rate.

**Figure 2.**
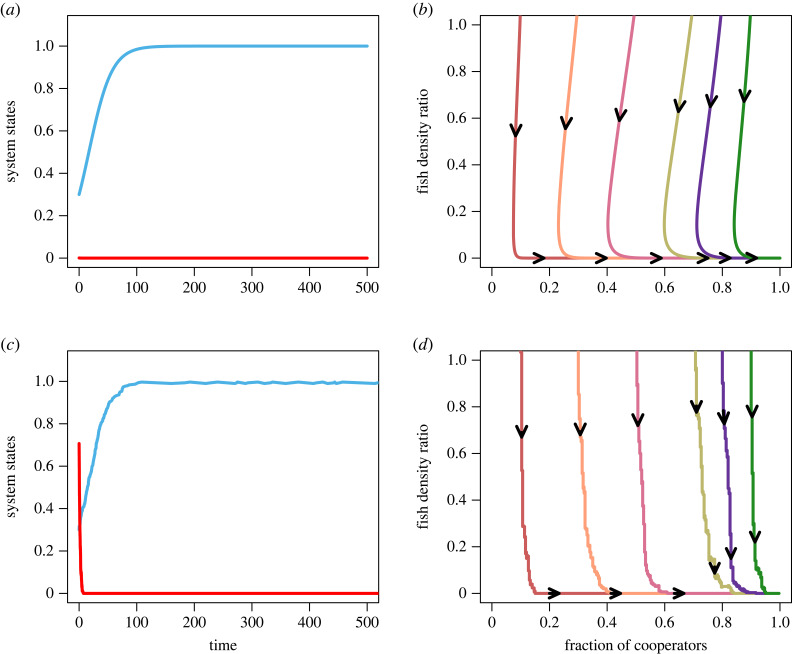
Temporal dynamics of co-evolutionary system in low growth rate. (*a*,*c*) Time series of the fraction of cooperators (cyan line) and the shared commons abundance (red line) for deterministic and stochastic systems respectively. (*b*,*d*) Phase portrait of *x*–*n*/*k* system. The distinct trajectories correspond to initial conditions (0.9, 1.1), (0.8, 1.1), (0.7, 1.1), (0.5, 1.1), (0.3, 1.1) and (0.1, 1.1). Cooperation is at its loftiest satisfactory level for *r* < *g*_*c*_ < *g*_*d*_; however, the low growth rate of resources makes resource abundance irrelevant. Parameters are *r* = 0.3, *N* = 1000, *k* = 1000, *α* = 0.7, *β* = 0.1, *b*_*m*_ = 0.5, *p*_1_ = 0.5, *p* = 0.5, *h* = 1.

First, we consider the case 0 < *r* < *g*_*c*_ < *g*_*d*_, which assumes that cooperators and defectors gain more than the resource growth. In particular, the resource is recovering with a low growth rate. In this situation, *r k* < *N b*_*m*_, the system has only one stable equilibrium (1, 0); in particular, the system exhibits tragedy of the commons (TOC). The deterministic and stochastic time series of the fraction of cooperators and the abundance of common resource is given in figure 2. It follows that at a low growth rate, cooperators always cooperate at a high level of satisfaction. The results further demonstrate that even if the punishment and inspection mechanisms are capable of driving the system to its full cooperator state, the system will still become fully depleted due to its limited intrinsic growth rate. This result indicates that the cooperation mechanism is not always enough to shape the coupled system. Managers must also keep the growth rate of resources in mind. If the resource is growing slowly, harvesters should behave differently and take less share. Otherwise, the shared commons are unable to recover, and extensive cooperation becomes futile.

### 2.6. Oscillatory dynamics in abate growth rate

**Figure 3.**
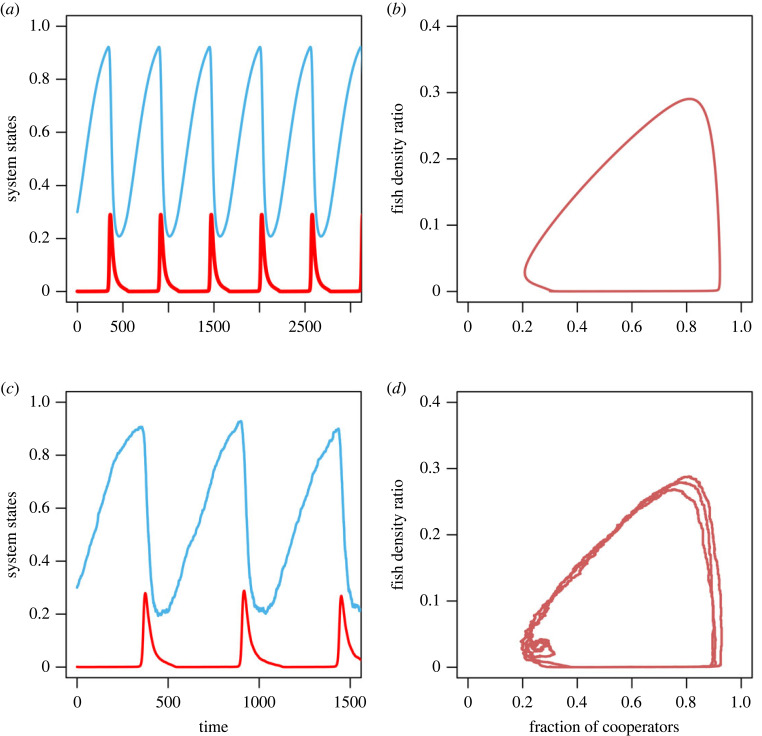
Persistent oscillations in co-evolutionary system for moderately growing commons. (*a*,*c*) Deterministic and stochastic time series of the fraction of cooperators (cyan line) and the shared commons abundance (red line). (*b*,*d*) Phase portrait dynamics of the coupled *x*–*n*/*k* system in both deterministic and stochastic systems. Parameters are *r* = 0.8, *N* = 1000, *k* = 1000, *α* = 0.7, *β* = 0.1, *b*_*m*_ = 0.5, *p*_1_ = 0.5, *p* = 0.1, *h* = 1.

When the growth rate of shared commons falls between the cooperators’ gain and defectors’ gain, means 0 < *g*_*c*_ < *r* < *g*_*d*_, then the outcomes are more interesting. In this case, we have *Nb*_*m*_ < *rk* < *Nb*_*m*_ (1 + *α*). We consider three different values for punishment and inspection mechanisms here since the institutional effect is better explained by the product of these two parameters *p* and *β* only. When *p* = 0.1 and *β* = 0.1, the system exhibits oscillation; the system exhibits oscillatory TOC (OTOC). The corresponding time evolution of the fraction of cooperators and the abundance of common resource is given in figure 3. It shows that for any initial condition *x*_0_ ∈ (0, 1) and *n*_0_ ∈ (0, 1), the global dynamics correspond to a closed periodic orbit (figure 3*b*,*d*). The dynamics demonstrate that if resource abundance is fully depleted, cooperators will enhance their cooperation level. Moderate growth will again increase the abundance of resources, then cooperators will decrease their cooperation rates due to low punishment and less inspection. In this way, the system exhibits periodic oscillations between high cooperation and defection and abundant resource resupply and depletion. Clearly, low punishment and a minimal inspection mechanism with a moderate growth rate are crucial to shaping a coupled system.

**Figure 4.**
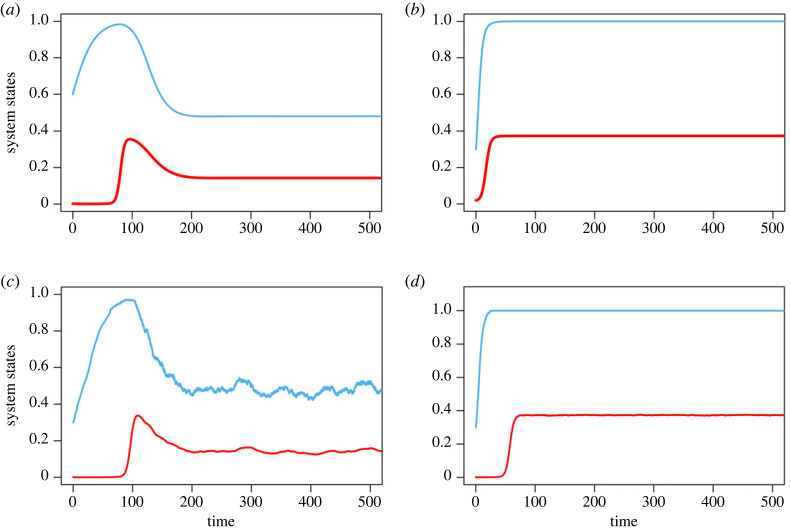
Temporal dynamics of co-evolutionary system for low punishment as well as strong punishment. (*a*,*b*) Deterministic time series of the fraction of cooperators (cyan line) and the abundance of risky commons (red line). (*c*,*d*) Stochastic dynamics of the *x*–*n*/*k* system. Parameters are for (*a*) *r* = 0.8, *N* = 1000, *k* = 1000, *α* = 0.7, *β* = 0.5, *b*_*m*_ = 0.5, *p*_1_ = 0.5, *p* = 0.1, *h* = 1, and for (*b*) *r* = 0.8, *N* = 1000, *k* = 1000, *α* = 0.7, *β* = 0.5, *b*_*m*_ = 0.5, *p*_1_ = 0.5, *p* = 0.5, *h* = 1.

If *p* = 0.1 and *β* = 0.5, the system has coexisting steady state: averted TOC. The corresponding deterministic and stochastic trajectories of the fraction of cooperators and the abundance of shared commons are given in figure 4*a*,*c*. It follows that at low levels of inspection, a reasonably strong external institution with moderate growth rates, cooperators and defectors will cooperate and defect simultaneously at a finite abundance of commons. Moreover, the results revealed that the severe punishment mechanism and low inspection mechanism have the capability of shaping the coupled system to its sustainable limit. This indicates that the system’s moderate intrinsic growth rate is important for a sustainable commons.

The system exhibits stable equilibrium in both states—averted TOC—if the probability of defection *p* = 0.5 and the punishment *β* = 0.5. This case is illustrated in figure 4*b*,*d*. In the presence of formidable external institutions with a moderate growth rate of shared commons, cooperators always cooperate at a high level of satisfaction. This indicates that all harvesters share the renewable commons cooperatively, and the system maintains the imperishable commons sustainability. The results of this study show that in moderately growing commons, high levels of inspection, along with strong punishment, can determine the shape of a coupled human–environment system.

### 2.7. Dynamical outcomes in high growth rate

**Figure 5.**
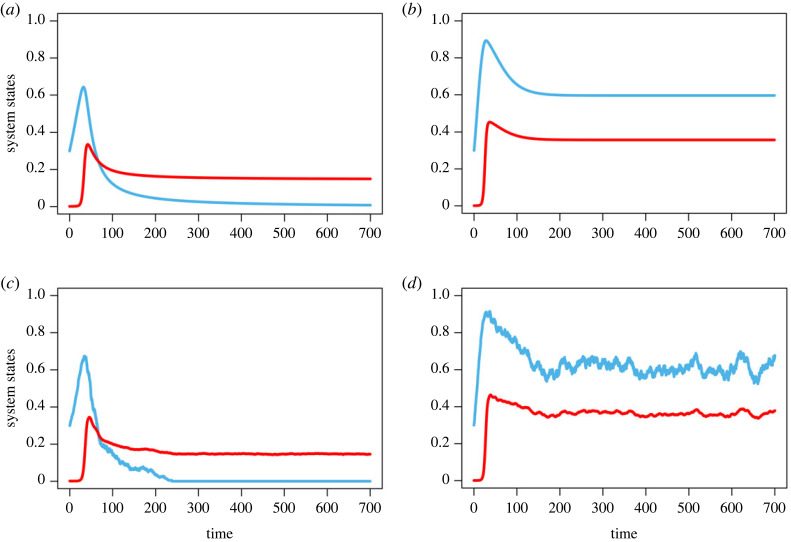
Replicator dynamics of co-evolutionary system for rapidly growing resource. (*a*,*b*) Deterministic time series of the fraction of cooperators (cyan line) and the abundance of risky commons (red line). (*c*,*d*) Stochastic dynamics of the coupled *x*–*n*/*k* system. (*a*,*c*) Parameters are *r* = 1, *N* = 1000, *k* = 1000, *α* = 0.7, *β* = 0.5, *b*_*m*_ = 0.5, *p*_1_ = 0.5, *p* = 0.1, *h* = 1. (*b*,*d*) Parameters are *r* = 1, *N* = 1000, *k* = 1000, *α* = 0.7, *β* = 0.25, *b*_*m*_ = 0.5, *p*_1_ = 0.5, *p* = 0.5, *h* = 1.

Finally, we consider 0 < *g*_*c*_ < *g*_*d*_ < *r*, that assumes both cooperators’ gain (*g*_*c*_) and defectors’ gain (*g*_*d*_) are smaller than the shared commons growth rate. In this situation, we have *r* > *N b*_*m*_ (1 + *α*) > *N b*_*m*_. Like previously discussed, here we consider two significantly distinct cases for punishment and inspection mechanisms. When *p* = 0.1 and *β* = 0.5, the system exhibits stable equilibrium in both states. The corresponding deterministic and stochastic temporal dynamics of the fraction of cooperators and the abundance of shared commons are illustrated in figure 5*a*,*c*. Accordingly, at low inspection and with a rapidly growing intrinsic growth rate, defectors always defect at a high level. Despite the commons existing non-trivially with finite abundance, the system will still converge to full defection due to its rapid intrinsic growth rate. Consequently, finite commons are not sufficient to maintain a sustainable human–environment system. In order to avoid defection, managers or institutions should look into participants’ behaviour. If the growth rate of the risky commons is high, harvesters should take an equal and much higher share from the resource pool. If not, finite renewable resources become useless. The system exhibits coexisting stable equilibrium if *p* = 0.5 and *β* = 0.25. This is illustrated in figure 5*b*,*d*. Furthermore, this demonstrates that cooperators and defectors can coexist in a strong external institution with a high growth rate. If the probability of defection *p* = 0.5 and punishment *β* = 0.5, the system exhibits stable equilibrium in both states. The corresponding dynamics is shown in electronic supplementary material, appendix figure S2. In high growth rate of shared commons, cooperators always cooperate at a high level of satisfaction. This indicates that high growth rates are crucial for sustainable resource distribution.

### 2.8. Richer dynamics

**Figure 6.**
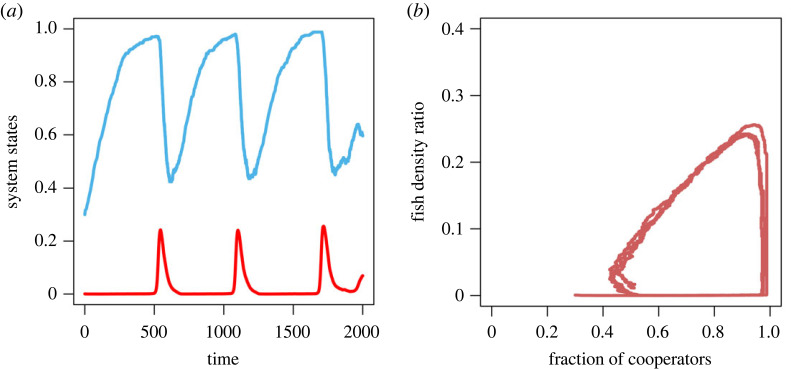
Persistent oscillations in co-evolutionary system for change in *p*_1_ and *r*. (*a*) Stochastic trajectories of the fraction of cooperators (cyan line) and the abundance of the risky commons (red line). (*b*) Stochastic phase plane dynamics of the coupled *x*–*n*/*k* system. Parameters are *r* = 0.7, *N* = 1000, *k* = 1000, *α* = 0.7, *β* = 0.1, *b*_*m*_ = 0.5, *p*_1_ = 0.391, *p* = 0.1, *h* = 1.

For certain parameter values of commons growth rate and predation, the coupled system of co-evolutionary games exhibits outcomes similar to those of high predation—OTOC. The corresponding stochastic time series of the fraction of cooperators and the abundance of shared commons is given in figure 6*a*. It shows that for any initial condition *x*_0_ ∈ (0, 1) and *n*_0_ ∈ (0, 1), the global dynamics correspond to a closed periodic orbit (figure 6*b*). The dynamics demonstrate that if shared commons abundance is fully depleted, cooperators will enhance their cooperation level. Low predation and growth will again increase the abundance of commons, then cooperators will increase their defection rates due to low punishment and less inspection. In this way, the system exhibits periodic oscillations between cooperation and defection and abundant commons resupply and depletion. Clearly, low predation with low punishment and a minimal inspection mechanism is crucial to shaping a coupled system.

**Figure 7.**
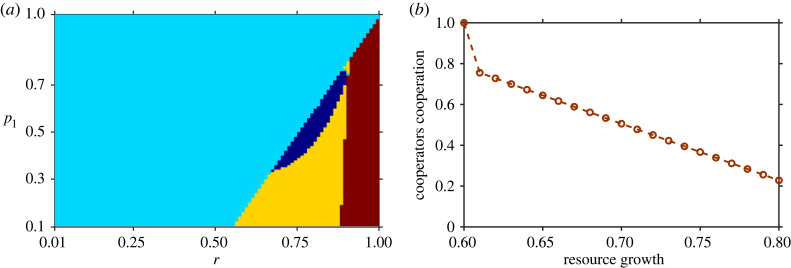
Summary of dynamics of co-evolving games for all possible combinations of *r* and *p*_1_. (*a*) Sensitivity analysis of two parameters. System exhibits four type of dynamics, tragedy of commons (TOC) (cyan), oscillatory TOC (blue), averted TOC (yellow) and full defection region (brown). (*b*) Cooperator cooperation level dynamics with resource growth rate *r*. Parameters are *N* = 1000, *k* = 1000, *α* = 0.7, *β* = 0.1, *b*_*m*_ = 0.5, *p*_1_ = 0.2, *p* = 0.1, *h* = 1.

In figure 7*a*, we have represented all possible dynamics using a phase plane. It shows that at low punishment and less inspection, with all possible combinations of *r* and *p*_1_, the system manifests four possible dynamics: TOC, OTOC, averted TOC meaning coexisting states or sustainable fisheries, and full defection. Also, the cooperators’ level of cooperation dynamics with the commons growth is shown in figure 7*b*. From a cooperation standpoint, it exhibits very interesting dynamics. It shows that when the predation rate is low (*p*_1_ = 0.2) and shared commons growth is high moderate (*r* = 0.8), the majority of harvesters become defectors with a low level of cooperative satisfaction because a high growth rate of commons prevents the system from establishing full cooperation (figure 7*b*).

However, this result is not true in general. For instance, very different outcomes occur when the growth rate is less moderate (*r* = 0.6) (figure 7*b*). Harvesters become very cooperative and cooperate with a high level of satisfaction because a lesser growth rate helps to build cooperation among harvesters. This, in turn, allows moderate fish density to be maintained and averts the TOC (*n*/*k* > 0 and stable) (figure 7*a*). Hence, with an increase in shared commons growth, harvesters decrease their cooperative nature and level of cooperation.

In addition, we also varied the growth rate *r* and the product of the probability of defection *p* and the punishment *β* (electronic supplementary material, appendix figure S1). Here also the system exhibits four possible dynamics. The corresponding temporal dynamics are also shown in electronic supplementary material, appendix figure S1. It shows that a low probability of defection and significantly less punishment with a moderate growth rate system exhibits OTOC. With high *pβ* and low growth rate *r*, the system has TOC, and with a high growth rate, the system exhibits averted TOC and a low level of cooperation.

## 3. Discussion

Recent studies have shown a growing interest in exploring the complexity of our world on many different levels using co-evolutionary game theory that incorporates feedback between environment and game and between game and environment [37,43]. Here, we developed a realistic model that couples the behaviour of participating players and the environment. This paper showed how a coupled co-evolving dynamical system manifests a richer variety of dynamical regimes, such as TOC, OTOC and full defection, than the uncoupled SES. Our analysis demonstrates how institutional conditions can cause changes in the multiple equilibria of an ecological system. Under institutional conditions such as moderate growth rate and predation, the feedback-evolving dynamical system may be managed to be fairly resilient to perturbations (random or consistent) that reduce the significance of tipping points. Low growth rate with low inspection and severe punishment increases the importance of the tipping point and decreases resilience. These insights emphasize that institutions can manage an SES and alter decisions to create a more sustainable evolutionary system. Many environmental issues stem from institutional failure, making this an important concept to consider [44].

In this more realistic model, we consider the fish populations that have Holling’s type II predation and harvesting. Here, we assess management approaches to limit overharvesting of fish populations. The central mechanism that underlies this study has been applied to both theoretical [45] and laboratory experiments [22,28], as well as realistic field studies, focused on forest ecosystem management [31,46]. This controlled approach assumes an effective institution that monitors harvesters. We have now shown that sustainable states are not only threatened by overexploitation. In particular, limiting our share is insufficient based on our common resource abundance. Instead, we must also consider those populations’ growing capacity, higher order nonlinear interactions, and predation. For example, strong and controlled mechanisms are unable to prevent TOC at low growth rates. On other extremes, high growth rates require the mentioned institutions only to stabilize the high cooperation level, but we can always maintain sustainable resource levels. Thus, we pointed out that punishing defectors and monitoring overexploitation have a critical role in the intermediate growth and predation rate of environmental resources.

Recreational fisheries are resilient enough, and it is widely accepted that they play a crucial role in establishing a sustainable system [47]. In fact, they are critical, but we must consider not only their temporal dynamics but also their intrinsic and extrinsic characteristics like nonlinear interactions, predation rate, and growth rate when designing their sustainable use [18]. Otherwise, our efforts to control the players in the co-evolutionary game become useless [28]. As a result, to address the issue of overexploitation in a SES, it is necessary to consider both the internal and external characteristics of the system. These features can predict whether the systematic mechanism is capable of increasing shared resource levels in the desired direction.

We could relax some assumptions in future work. Different factors can also affect the correspondence between fish biomass and the fraction of cooperators. For instance, group size plays a key role in successfully managing shared resources [31]. We do not explicitly account for the influence of group size, such as small groups are most effective and can pronouncedly impact dynamics in a way that merits mechanistic modelling. Our model does not account for the impact of slow and fast time scales. Studies have shown that human behaviours are on fast time scales compared to shared resource dynamics, which the present model does not consider. As a result, future work could examine the changing speed between resource dynamics and population cooperation levels.

Future research could also develop more sophisticated control mechanisms to apprehend the dynamics of co-evolution between fisheries and human cooperation. Despite these concerns, these results may be useful for designing control mechanisms of punishment for regulating the risky commons by understanding how growing capacity, predation, and nonlinear interactions between intrinsic and extrinsic factors contribute to the growth of renewable resources.

## Data Availability

The data are provided in electronic supplementary material [48].
